# Trends in per-operative parameters and postoperative complications associated with coronary artery bypass graft surgery (CABG); A four-year retrospective study

**DOI:** 10.12669/pjms.37.7.4315

**Published:** 2021

**Authors:** Azam Jan, Muhammad Khizar Hayat, Mohammad Ahmed Arsalan Khan, Rafi Ullah

**Affiliations:** 1 Dr. Azam Jan, MD, Diplomate American Board of General Surgery (USA), Diplomate American Board of Thoracic (Cardiothoracic) Surgery (USA) Department of Cardiothoracic & Vascular Surgery, Rehman Medical Institute (RMI), Peshawar, Khyber Pakhtunkhwa, Pakistan; 2 Dr. Muhammad Khizar Hayat, MBBS Department of Cardiothoracic & Vascular Surgery, Rehman Medical Institute (RMI), Peshawar, Khyber Pakhtunkhwa, Pakistan; 3 Dr. Mohammad Ahmed Arsalan Khan, MBBS Department of Cardiothoracic & Vascular Surgery, Rehman Medical Institute (RMI), Peshawar, Khyber Pakhtunkhwa, Pakistan; 4 Dr. Rafi Ullah, MD. Department of Cardiothoracic & Vascular Surgery, Rehman Medical Institute (RMI), Peshawar, Khyber Pakhtunkhwa, Pakistan

**Keywords:** Coronary Artery Bypass, Intensive care units, Cardiothoracic Surgery

## Abstract

**Objective:**

To determine the patterns of per-operative parameters and early outcomes of patients that underwent CABG surgery during a four-year period.

**Methods:**

This is a cross-sectional descriptive study conducted in a tertiary care of hospital from November 2020 to January 2021. All the patients that underwent the isolated coronary artery bypass grafting (CABG) procedure were included in the study from June 2017 till June 2020. Data was collected on a data extraction form and stored in SPSS format which was analyzed for qualitative statistics keeping p<0.05 as significant. All the results were represented in the form of tables.

**Results:**

A total of 1,613 patients were operated upon for Coronary Artery Bypass Grafting (CABG) procedure during the study period with 1,222 (75.8%) males and 391 (24.2%) females. Dyslipidemia (71.8%) was the most common risk factor. The average perfusion time decreased only slightly (~1 minute) from 96.01 minutes to 95.07 minutes (2017 to 2020). This change however was not significant (p=0.301). The rate of Left Internal Mammary Artery (LIMA) use stayed relatively stable over the 4-year period fluctuating between 88.7% and 92.9% (p=0.360). The average initial ICU stay (in hours), drain at 12 hours and 24 hours stays almost the same. The rate of mortality peaked in 2018 (4.76%) and subsequently fell to 3.57% by 2020.

**Conclusion:**

More males underwent CABG surgery at this tertiary care hospital and the overall complication rate and per-operative parameters improved over the years. The non-risk stratified mortality in this study was found to be higher than developed nations.

## INTRODUCTION

Coronary Artery Bypass Grafting (CABG) is a common surgical procedure, representing annual volumes of approximately 200,000 cases in the United States and an average incidence rate of 62 per 100,000 inhabitants in Western European countries.^1^ People with severe Coronary artery disease undergo CABG in order to improve blood flow to the heart as it is one of the most effective treatment against blocked coronary arteries and the complications the blockage may cause. This management plan carries a per-operative mortality of approximately 2% while other complications range between 5% and 7%.^2^

In this procedure a healthy artery or vein from the body is connected, or grafted, across the blocked coronary artery. The grafted artery or vein bypasses the blocked portion of the coronary artery and restores blood flow to the ischemic area. This procedure hence reduces the mortality in patient with severe coronary artery disease.^3^

A range of complications can occur in patients undergoing CABG surgery. This includes atrial fibrillation (30%), Vasoplegia (20.3%), Stroke (5%), Mediastinitis (5%), Death (1-2%), Pulmonary hypertension (2%) and Renal dysfunction (21.3%).^4^-^7^ Other less common complications also occur. In this study, we determine the patterns of per-operative parameters and early outcomes of patients that underwent CABG surgery during a four-year period.

## METHODS

This is a cross-sectional descriptive study conducted in a tertiary care hospital of Peshawar from November 2020 to January 2021. All the patients that underwent the isolated coronary artery bypass grafting (CABG) procedure were included in the study from June 2017 till June 2020. Any additional surgeries with CABG, i.e., CABG plus ASD, VSD or valve replacement were excluded from the data however, CABG with endarterectomy and CABG with LV plication were included.

Pre-operatively, all the patients were assessed according to the AHA/ACC guidelines, decision for surgery were taken by multidisciplinary team which included cardiologists, echocardiographers, general physicians and cardiac surgeons. Per-operatively, LIMA was not used if LIMA flow was not adequate intra-operatively and surgeon’s preference in some emergencies or if the patients were old.

Data was retrieved from the Cardiothoracic Surgery ward archives which was collected on data extraction forms. The data collected was then exported in Microsoft Excel format which was then converted into SPSS format. It was analyzed using SPSS for qualitative statistics keeping p<0.05 as significant. All the results were represented in the form of tables.

### Ethical Approval

(Ref: RMI/RMI-REC/Article Approval/52, Dated: February 16, 2021)

## RESULTS

A total of 1,613 patients were operated upon for Coronary Artery Bypass Grafting (CABG) procedure during the study period with 1,222 (75.8%) males and 391 (24.2%) females. The mean age of patients was 58.03 ± 9.07 years with a minimum age of 25 years and maximum age of 85 years. The average weight and height were 74.10 ± 13.31 kg and 163.80 ± 10.05 cm respectively.

The risk factors in CABG patients is shown in Table-I. The most common of the risk factor was dyslipidemia (71.8%) followed by Hypertension (64.9%) and Diabetes (42.7%). Although a known risk factor, smoking only accounted for 9.3% of the patients that underwent CABG surgery.

**Table I:** Risk Factors of CABG Patients (N=1613).

*Factor*	*N (%)*
Diabetes	688 (42.7)
Hypertension	1047 (64.9)
Smoking/Tobacco Use	157 (9.7)
Family History of CAD	90 (5.6)
Dyslipidemia	1158 (71.8)
Cerebrovascular Disease	83 (5.1)
Dialysis Dependent	5 (0.3)
Chronic Lung Disease	36 (2.2)
Infective Endocarditis	44 (2.7)
Rheumatic Fever	2 (0.1)
Carotid Artery Disease	69 (4.3)

The per-operative parameters of CABG during the four-year period is shown in Table-II. The average perfusion time decreased only slightly (~1 minute) from 96.01 minutes to 95.07 minutes (2017 to 2020), peaking in 2019 (98.12 minutes). This change however was not significant (p=0.301). The aortic cross clamp time (CCT) was least in 2017 (52.89 ± 14.55 minutes) but increased over the following two years. In 2020, the CCT dropped again but was still only just higher than the value in 2017 (p=0.538). The rate of Left Internal Mammary Artery (LIMA) use stayed relatively stable over the 4-year period fluctuating between 88.7% and 92.9% (p=0.360). It can be seen that majority of patients received three grafts, ranging between 90% and 95%.

**Table II:** Per-operative Parameters.

Parameter		Years				P-value

		*2017 (N=382)*	*2018 (N=588)*	*2019 (N=502)*	*2020 (N=140)*	
Perfusion Time (min)		96.01 ± 25.36	95.58 ± 22.68	98.12 ± 24.84	95.07 ± 26.79	0.301
Cross Clamp Time (min)		52.89 ± 14.55	53.11 ± 15.10	54.21 ± 15.82	52.91 ± 16.16	0.538
LIMA Use		339 (88.7%)	523 (88.9%)	457 (91.0%)	130 (92.9%)	0.360
Number of Grafts*	1	4 (1.1)	3 (0.5)	3 (0.6)	3 (2.1)	-
	2	15 (3.9)	38 (6.5)	33 (6.6)	11 (7.9)	
	3	363 (95.0)	547 (93.0)	466 (92.8)	126 (90.0)	

* Data for 1 patient missing.

The Intensive Care Unit (ICU) parameters after CABG surgery is shown in Table-III. The average initial ICU stay (in hours), drain at 12 hours and 24 hours stays almost the same. The initial hours on ventilator saw a rise over the years.

**Table III:** Intensive Care Unit (ICU) Parameters.

Parameter	Years				P-value

	*2017 (N=382)*	*2018 (N=588)*	*2019 (N=502)*	*2020 (N=140)*	
Initial ICU Stay (hrs)	49.83 ± 13.46	50.27 ± 14.96	50.83 ± 19.78	51.92 ± 22.35	0.601
Drain at 12 Hr (ml)	421.58 ± 292.43	452.22 ± 367.23	441.27 ± 378.76	490.96 ± 439.17	0.253
Drain at 24 Hr (ml)	590.16 ± 357.07	622.85 ± 450.55	615.03 ± 556.52	664.86 ± 441.14	0.367
Initial Hrs Ventilated (hrs)	5.70 ± 7.36	6.22 ± 9.27	5.88 ± 9.35	6.57 ± 10.76	0.703

The post-operative parameters during hospital stay are sown in Table-IV. The rate of mortality peaked in 2018 (4.76%) and subsequently fell to 3.57% by 2020. In 2017, re-opening after bleeding tamponde (5.76%) was the most common complication and this trend continued over the years, highest (10.0%) in 2020. Atrial fibrillation (0.71 – 2.88%) was the post-operative complication to see the highest drop. Only one patient went into coma (2010) and in the same year two patients required dialysis. Over the years, it was seen that patients required prolonged ventilation (1.83-2.86%) and were re-intubated the highest in 2017 (1.31%). Ventricular Tachycardia was not seen as a complication in 2020, amongst several others.

**Table IV:** Post-operative Parameters, N (%).

Parameter*	Years				P-value

	*2017 (N=382)*	*2018 (N=588)*	*2019 (N=502)*	*2020 (N=140)*	
Re-intubated during hospital stay	5 (1.31)	7 (1.19)	5 (1.00)	-	0.604
Re-opening for bleeding tamponade	22 (5.76)	32 (5.44)	22 (4.38)	14 (10.0)	0.086
Deep Sternal Wound Infection	1 (0.26)	1 (1.70)	4 (0.80)	-	0.291
Post-op Stroke >72Hrs	2 (0.52)	3 (0.51)	8 (1.60)	-	0.109
Transient Neurological Deficit	-	2 (0.34)	3 (0.60)	-	0.394
Coma (>24 Hrs)	-	-	1 (0.20)	-	0.529
Dialysis Required	1 (0.26)	-	2 (0.40)	-	0.441
Prolonged Ventilation (>24Hr)	7 (1.83)	14 (2.38)	10 (2.00)	4 (2.86)	0.870
Atrial Fibrillation	11 (2.88)	11 (1.87)	7 (1.39)	1 (0.71)	0.287
Ventricular Tachycardia	3 (0.79)	3 (0.51)	3 (0.60)	-	0.758
Mortality	13 (3.40)	28 (4.76)	20 (3.98)	5 (3.57)	0.742
Discharged	369 (96.60)	560 (95.24)	482 (96.02)	135 (96.43)	

* Data for 1 patient missing.

## DISCUSSION

Despite multiple randomized clinical trials in the last several decades, the ideal treatment option for patients with multivessel CAD is still uncertain. A recent trail published in 2020, comparing CABG, PCI and Hybrid coronary revascularization have stated residual myocardial ischemia and major adverse cardiovascular and cerebral events (MACCE) post-procedurally.^8^ The dilemma therefore remains unresolved. The choice between CABG and PCI in patients with multivessel (two- and three-vessel) disease is influenced by a number of factors, including the number of vessels involved, the amount of myocardium supplied by the affected vessels, the anatomic complexity of the lesions requiring revascularization, likelihood of complete revascularization, patient comorbidities such as diabetes, and patient preference. At the time of decision making, the patient should be informed about the relative risks of death, stroke, and the need for repeat revascularization.

When the coronary anatomy is equally well suited for both PCI and CABG, the decision should be made jointly by the patient, referring physician, cardiologist, and a heart team or cardiothoracic surgeon. Attention must be paid to the patient’s expectations and willingness to undergo repeat procedures, if necessary. Some patients remain fearful of open heart surgery with increased periprocedural morbidity and mortality and the ensuing 4- to 12-week convalescence, while others are unwilling to undergo repeat interventions if necessary. Other patients are strongly stroke averse and are willing to accept an increased risk of revascularization after PCI, in order to decrease the increased risk of stroke with CABG. The importance of clinical decision making after consideration of all of these factors should not be underestimated and cannot be easily replaced even by the best evidence from observational or randomized clinical trials.

In this study, CABG was predominantly performed on male (75.8%) patients. This trend is seen in multiple studies with men accounting for majority of the surgeries. Other studies show a range of 60-70% male predominance.^9^-^11^ The average age of patients in this study was also near 60 years with an 85-year-old also operated. The increased safety of the surgery can be a factor in such a trend.^12^

Comparing the characteristic risk factors, it can be seen that Hypertension is the leading risk factor among the American and Chinese population (78.4% and 58.4% respectively). This is closely followed by Smoking (52.7%) in Chinese and Hyperlipidemia (59.5%) in American population according to a study by Zheng et.al.^13^ In our study, dyslipidemia (71.8%) followed by Hypertension (64.9%) and Diabetes (42.7%) were the leading factors. In a study conducted in Pakistan, Diabetes (60%) was found to be leading risk factor. Smoking (23%) was the 3^rd^ leading risk factor however, it was not a significant risk factor in our study (9.3%).^14^ Hypertension (41.0%) and Diabetes (32.1%) were also contributors in another study.^15^

Use of LIMA in CABG procedures have become a standard practice nowadays. The convenience of anastomosis (only distally), satisfactory rate of patency, long-term survival of patients and decreased mortality rate have made it an asset to CABG procedures. This has been emphasized in various studies.^16^-^17^ Hence, the LIMA was preferentially used in our setup with success rates of 92.9% in 2020. The success rate in 2020 is the highest overall in comparison to that of the previous years. This can be attributed to the lower volume of surgeries in 2020 due to the ongoing COVID-19 pandemic. However, the overall LIMA success rate in our setup is below par in comparison with other international studies, 94.6% in a study conducted in Turkey, 100% in the United Kingdom and 96.4% (off-pump) and 93.2% (on-pump) in another center in Turkey.^5^-^7^ No statistical difference of note was seen between the year groups (p=0.360). The reason for low rates of LIMA success can be owed to the late presentation of patients in our setup for surgery.

Many complications, per-operative, intraoperative and post-operative, have been associated with this procedure, some are discussed below.

Atrial fibrillation (AF) is the most common sustained arrhythmia encountered in clinical practice and one of the common and morbid complication following cardiac surgery. Its incidence after cardiac surgery is 35% while it approximately affects 1% of the total population and 8% of individuals over 80 years old.^4^ Due to the enhancement of surgical techniques and increasing quality of post-operative care, elderly patients with increased arrhythmia risk undergo cardiac revascularization which further increases the incidence of post-operative atrial fibrillation (POAF). In the present paper however, the incidence of POAF is considerably low, 0.71% in 2020. This is the most recent percentage, the highest being in 2017 (2.88%). Other studies conducted throughout the world put the incidence of POAF at 20-30%.^5^-^7^ One study conducted by Khalifa et al, showed POAF to be 6% which corresponds with our study albeit slightly higher.^18^ The reason for such a low rate of AF in our study pertains to the prophylactic use of beta blockers in our patients. Care in taken when prescribing beta blockers and is substituted with calcium channel blockers or digoxin in cases in which the beta blockers are contraindicated. As shown, this practice significantly reduces the rate of AF.

Deep sternal wound infection (DSWI) is an uncommon complication for patients who have undergone cardiac surgery. However, the patients that do present with DSWI are at an increased risk of mortality, 6-30%.^19^ In a 10-year surveillance study of sternal wound infections, methicillin-sensitive Staphylococcus aureus accounted for 28.3% of the isolates, Pseudomonas aeruginosa 18.3%, methicillin-resistant Staphylococcus aureus 14.6%, and Enterobacter species 6.7%.^20^ In our study, 2020 did not see any DSWI however, from 2017 to 2019, the DWSI rates ranged between 0.26% to 1.70%. The mainstay of treatment is a pus swab culture and culture-directed antibiotic therapy with regular dressing. Empirically, broad spectrum beta-lactam combined with Vancomycin and an aminoglycoside can be administered.^21^ Debridement and negative pressure wound therapy can also be considered upon consultation.

Post-operative stroke risk is increased if CABG is performed early after an acute myocardial infarction however, the incidence markedly decreases if CABG is performed later. In a study published by scientific reports, 3.7% of the patients presented with an ischemic stroke within 90 days.^22^ In our study the highest incidence is 1.60% and 0.60% for a transient ischemic attack.

The in-hospital mortality rate of China (1.2%) and United States (1.3%) are significantly lower as compared to those of our center (3.40%, lowest amongst four years).^13^ In a study by Saxena et.al, the in-hospital male mortality (1.6%) and female mortality (1.8%) were also found to be considerably low.^23^ The ASCERT study linked data from the Society of Thoracic Surgeons Adult Cardiac Surgery Data base and the Centers for Medicare and Medicaid Services (United States). It included around 350,000 isolated CABG patients whose Kaplan Meir mortality was estimated to be 3.2% at 30 days, 6.4% at 180 days, 8.1% at one year, 11.3% at two years, and 23.3% at three years of follow- up. Using registry data in the United States, the perioperative and in-hospital mortality rate after coronary artery bypass graft surgery (CABG) between 1997 and 2001 averaged about 1% for the lowest risk elective patients, and 2 to 5% for all patients.^24^ This corresponds to our study. Different factors that influence the mortality need to be taken into account. Such factors include the patients age, co-morbidities, gender and stage of presentation. Patients mostly due to financial constrains or difficulty in travel tend to present to the tertiary care center late.

### Limitations of the study

The major limitation to this study is the retrospective nature of the data collection. This being a single center study is another limitation. Only pre-discharge data was used for analysis hence only outcomes at discharge could be analyzed while short and long-term outcomes could not be determined. A study with long-term follow-up should provide a clearer picture of the outcomes.

## CONCLUSION

More males underwent CABG surgery at this tertiary care hospital and the overall complication rate and per-operative parameters improved over the years. The non-risk stratified mortality in this study was found to be higher than developed nations.

### Authors Contribution:

**AJ:** Conceived, and designed.

**MKH, MAAK & RU:** Did manuscript writing.

**MKH:** Did statistical analysis & editing of manuscript.

**AJ, MAAK & RU:** Did data collection and generation of data bank.

**AJ & MKH:** Did review, final approval of the manuscript & are accountable for the accuracy and integrity of the work.
